# Self-assembled materials and supramolecular chemistry within microfluidic environments: from common thermodynamic states to non-equilibrium structures

**DOI:** 10.1039/c8cs00025e

**Published:** 2018-05-01

**Authors:** S. Sevim, A. Sorrenti, C. Franco, S. Furukawa, S. Pané, A. J. deMello, J. Puigmartí-Luis

**Affiliations:** a Institute for Chemical & Bioengineering , Department of Chemistry & Applied Biosciences, ETH Zurich , Zurich 8093 , Switzerland . Email: josep.puigmarti@chem.ethz.ch ; Email: alessandro.sorrenti@chem.ethz.ch; b Institute for Integrated Cell-Material Sciences (WPI-iCeMS) , Kyoto University , Yoshida , Sakyo-ku , Kyoto 606-8501 , Japan; c Multi-Scale Robotics Lab (MSRL) , Institute of Robotics & Intelligent Systems (IRIS) , ETH Zurich , Zurich 8092 , Switzerland

## Abstract

Self-assembly is a crucial component in the bottom-up fabrication of hierarchical supramolecular structures and advanced functional materials. Control has traditionally relied on the use of encoded building blocks bearing suitable moieties for recognition and interaction, with targeting of the thermodynamic equilibrium state. On the other hand, nature leverages the control of reaction–diffusion processes to create hierarchically organized materials with surprisingly complex biological functions. Indeed, under non-equilibrium conditions (kinetic control), the spatio-temporal command of chemical gradients and reactant mixing during self-assembly (the creation of non-uniform chemical environments for example) can strongly affect the outcome of the self-assembly process. This directly enables a precise control over material properties and functions. In this tutorial review, we show how the unique physical conditions offered by microfluidic technologies can be advantageously used to control the self-assembly of materials and of supramolecular aggregates in solution, making possible the isolation of intermediate states and unprecedented non-equilibrium structures, as well as the emergence of novel functions. Selected examples from the literature will be used to confirm that microfluidic devices are an invaluable toolbox technology for unveiling, understanding and steering self-assembly pathways to desired structures, properties and functions, as well as advanced processing tools for device fabrication and integration.

## Abstract 2

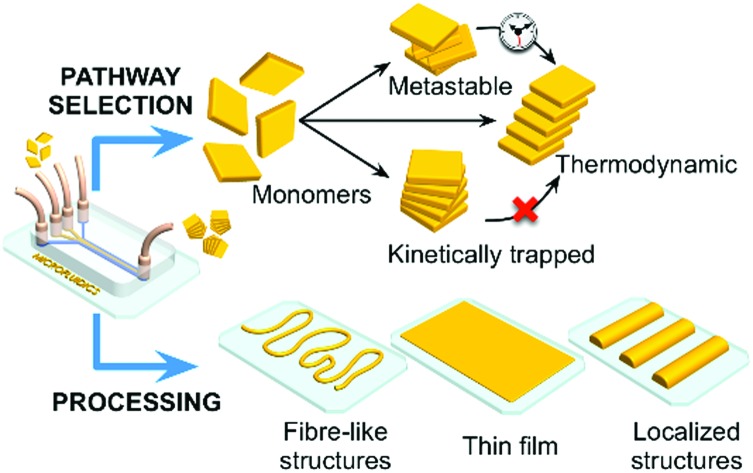
Microfluidics enables selection of different pathways in self-assembly processes, while allowing for an exquisite control over the processing of self-assembled materials.

##

Key learning points

(1) Microfluidic technologies offer unique physical conditions.

(2) There are striking differences between flask mixing experiments and controlled diffusion mixing under laminar flow conditions.

(3) Why out-of-equilibrium conditions are of high interest in chemistry, physics and materials science.

(4) Microfluidic technologies are advanced tools to isolate and study non-equilibrium assemblies and for processing of materials on surfaces.

(5) Microfluidic technologies enable one to study chemical and physical phenomena that cannot be investigated and resolved by other methods.

## 1. Introduction

Over the past three decades, supramolecular chemistry and self-assembly processes have attracted much attention due to their key roles in the bottom-up fabrication of micro and nanostructures and the preparation of materials with rationalized properties and functions.^1^ In chemistry, supramolecular assemblies are commonly considered the end result of the self-assembly of “encoded” molecular components,^2^ yet the chemical and physical pathways (in conjunction with the environment) that make such processes possible are rarely understood and often the subject of only speculation and observation.^3^ Traditional control over self-assembly has relied on careful molecular design, where specific moieties are incorporated into the constituent units that direct molecular recognition and interaction. Functional entities, such as amine, amide, carboxyl, or hydroxyl groups, can spontaneously interact and generate well-defined assemblies through covalent and non-covalent interactions. However, such assemblies cannot be further modified to provide diverse morphologies and/or functions. Put simply, although a chemist is able to change the chemical reactivity of molecules and the way such molecules interact, the products of the reaction may not necessarily be the ones desired.

On the other hand, living systems create hierarchically organized materials with functions of remarkable complexity, controlling the self-assembly of individual components in both time and space. Additionally, such self-assembled structures are dynamic, in that they can change (or adapt) their organization and morphology in response to an environmental or chemical cue. Surprisingly, these dynamic processes proceed in a self-regulating manner *via* control of chemical species diffusion or through a rational supply of external energy inputs.^4^ It has been demonstrated that in nature many bio-chemical processes (leading to complex and sophisticated biological functions) occur *via* control over the chemical species diffusion. For example, the diffusion of intracellular calcium atoms has a regulating effect on vital cellular functions, including motility, cell growth, or fertilization.^5^ Accordingly, such reaction–diffusion (RD) processes found in nature should be a source of inspiration to experientialists in terms of gaining new insights regarding the preparation of self-assembled functional materials.^6^

Recently, van Esch *et al.* have shown how controlled RD processes can be used to prepare free-standing structures with defined shapes, sizes, and functions.^7^ In this study, the authors show how molecular self-assembly can be locally controlled through the diffusion of two or more reagents within a hydrogel matrix that acts as a reaction substrate (where the hydrogel matrix regulates and adjusts reagent diffusion). The results presented are of particular interest for the fabrication of rationalized 3D microstructures, the preparation of chemical gradients and the spatial control of material composition. However, to lay the foundations of new developments in supramolecular chemistry, new technologies that facilitate extraordinary control of reagent (or chemical stimuli) diffusion are needed.^8^ Indeed, the spatio-temporal command of reactant mixing during self-assembly can enable an exquisite control over material properties, even allowing the isolation of intermediate states and unprecedented structures and/or the emergence of novel functions. Additionally, the isolation of intermediate states (or kinetically trapped structures) may also help to shed light on the pathway complexity underlying self-assembly processes in general, eventually leading to both their evaluation and adjustment. As a consequence of improved kinetic control and understanding of self-assembly pathways, tailored assemblies with bespoke properties and functions are within reach.

**Fig. 1:**
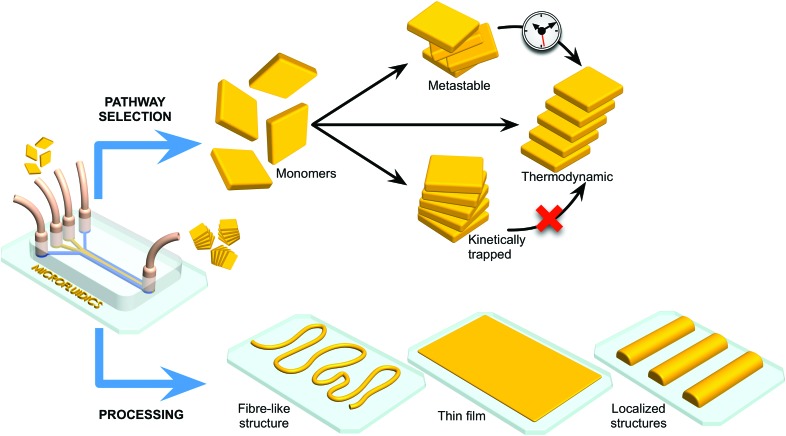
Microfluidic technologies enable selection of different pathways in self-assembly processes, yielding structurally different assemblies and/or thermodynamic states at either thermodynamic equilibrium or non-equilibrium states (*i.e.* metastable, slowly relaxing to equilibrium, or kinetically trapped structures). At the same time, the microfluidic approach allows for exquisite control over the processing of self-assembled materials on surfaces, *e.g.* enabling direct printing of fibre-like structures, formation of thin films, and formation of localized patterns.

In the context of the above discussion, microfluidic technologies have been shown to be particularly adept at controlling reactive processes (Fig. 1). For example, the laminar flow regime characteristic of most microfluidic devices allows for a fine control over mass transfer rates in both time and space,^9^ with mixing being solely mediated by diffusive processes. This is in sharp contrast to the conditions representative of conventional batch syntheses. In such environments, the diffusion of reagents cannot be easily controlled, with turbulence ensuring rapid mixing of reagents. Apart from their ability to control diffusion, microfluidic technologies offer other unique features, such as a large surface-to-volume ratio, enhanced mass and heat transfer, and the ability to compartmentalize reactions in small volumes (Table 1).

**Table 1:** Unique conditions offered by microfluidic environments and their effect in self-assembly and supramolecular processes

Conditions	Effect
Laminar flow: mixing only through molecular diffusion (no turbulence mixing)	Enhanced control over the reaction–diffusion area and the residence time of self-assembly processes; fine-tuning of the ordering of reagents in time and space (spatiotemporal control of reaction stages); time-controlled multi-step reactions
(If the reaction process is faster than mixing)	Possibility to influence the selection pathway (assembly); isolation of kinetically trapped assemblies; generation of target intermediates not accessible with bulk-type methodologies
Free interface diffusion	Favourable for homogeneous and single nucleation events; slow rate of mixing; a way to reach critical supersaturation; rapid screening of reaction conditions due to the slow rate of mixing
Controlled supersaturation	High supersaturation: increased nucleation rate, small assemblies
	Low supersaturation: homogeneous nucleation
Large surface-to-volume ratio	Enhanced nucleation (crystallization)
Excellent heat and mass transfer	Immediate changes in temperature; isothermal conditions; rapid or slow mixing of reagents; accelerated reaction speeds and efficiencies
Free-convection environment	Favourable for homogeneous nucleation events; robust conditions for the investigation of self-assembly and supramolecular processes
Compartmentalization of small volumes (pl–μl)	Ideal for parallel screening of various conditions in a reduced area; control over reagents’ concentrations and supersaturation

Unsurprisingly, a great deal of attention has been focused on the use of microfluidic devices as advanced tools for the preparation of new materials and assemblies. For example, Cronin and co-workers have recently described the isolation of new intermediate polyoxometalate (POM) structures using extremely simple microfluidic tools. Briefly, the authors leveraged microfluidics as a versatile tool for performing mechanistic studies of POM self-assembly and engineering new nanoscale POM structures in a continuous and robust manner.^10^ The latter aspect is of particular relevance since POMs are commonly considered as key inorganic clusters for applications in catalysis, medicine, and nanotechnology.^10^ Additionally, other researchers have used microfluidic devices to prepare a range of different materials, including multifunctional and multicomponent nanostructures that are unattainable with conventional flask stirring experiments.^11^ Ensembles of micro-droplets formed in 2D-microfluidic devices have also been used as valuable tools for studying non-equilibrium phenomena and long-range hydrodynamic interactions, as recently reviewed.^12^ It is therefore unsurprising that microfluidic technologies have witnessed an increasingly rapid growth and acceptance in chemistry, materials science and physics.^13^ Lastly, as shown in Fig. 1, microfluidic devices can also be used to control self-assembly on surfaces, and function as advanced processing tools for device fabrication and integration.

Herein, we attempt to summarize and examine selected studies that showcase the impact and potential of microfluidic technologies in the fields of self-assembled materials (Section 2) and supramolecular chemistry (Section 3). We will show how the unique features of microfluidic flows have been advantageously employed to control the self-assembly of materials and to steer pathway selection in non-covalent supramolecular processes (*e.g.* for the isolation of metastable, kinetically trapped or thermodynamic assemblies).^14^ In the latter case, discrete supramolecular aggregates can be studied and characterized in a direct fashion using optical and/or spectroscopic methods (*vide infra*). Finally, we provide an outlook of the general field, highlighting challenges and future opportunities.

## 2. Processing of self-assembled materials under microfluidic conditions

The properties and functions of self-assembled materials are intrinsically associated with their assembly and spatial arrangement (orientation) at the molecular level: features that ultimately define their field of application and performance. Accordingly, it is reasonable to assume that controlling self-assembly processes of materials (*e.g.* from amorphous to crystalline) is essential in providing access to a variety of unprecedented functional structures and new applications. Significantly, the integration of functional materials with read-out components located on a surface is an indispensable step in achieving fully operational systems and devices. In this section, we show how microfluidic technologies have been used for the controlled synthesis of self-assembled materials in solution and on surfaces, with the main focus on the formation of micro-sized materials.

### Solution-based syntheses

#### Organic-based conductors

**Fig. 2:**
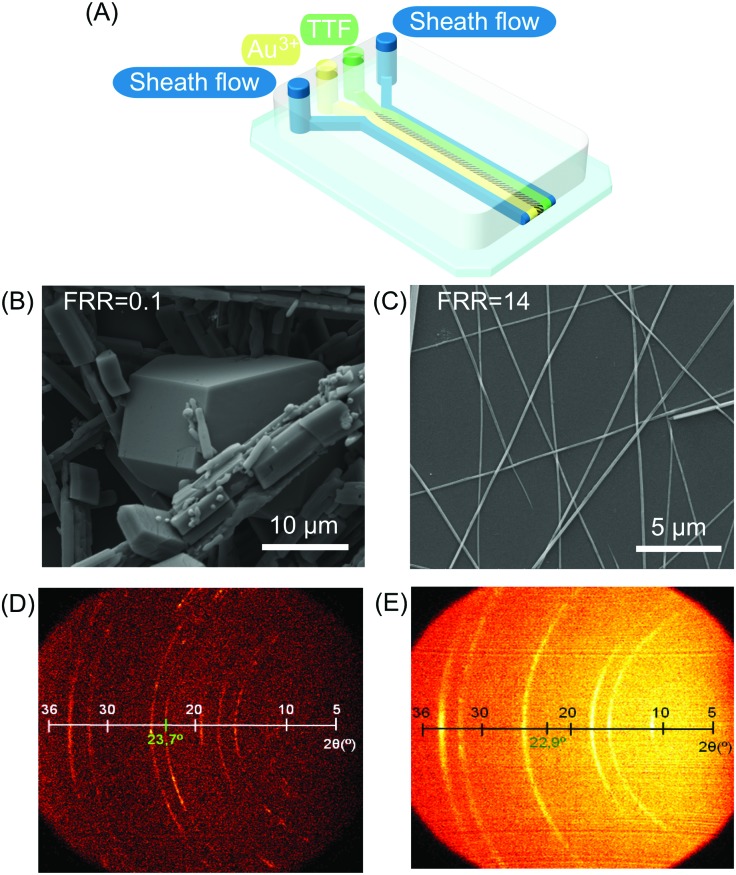
(A) Schematic illustration of the microfluidic device for TTF assembly. (B and C) Scanning electron microscopy (SEM) images of TTF-Au structures collected outside the chip and synthesized at a FFR of 0.1 and 14, respectively. (D) and (E) Bi-dimensional X-ray images of samples prepared with different FRRs (10 and 14 respectively). The green numbers indicate the reflexion peak related to the π–π stacking distance between TTF molecules in the generated structures. Reproduced from ref. 19 with permission from Wiley-VCH Verlag GmbH & Co., copyright 2010.

**Fig. 3:**
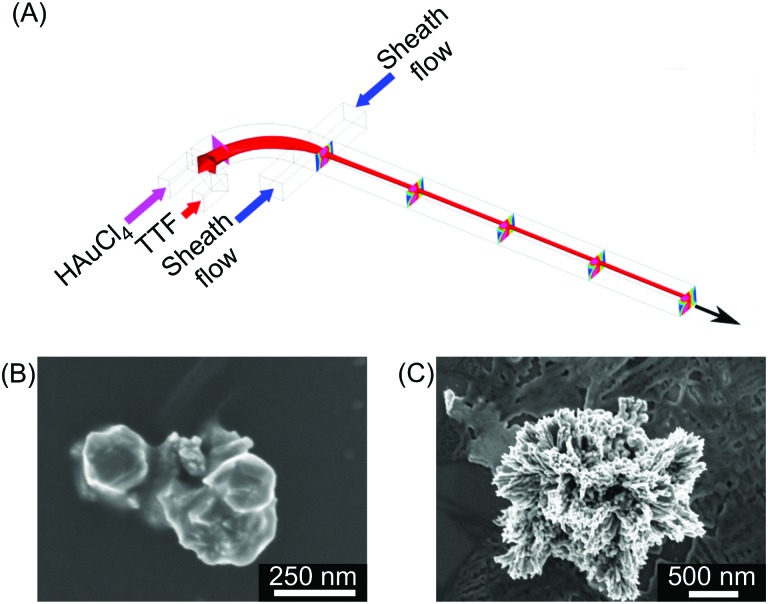
(A) Schematic drawing of the 3D hydrodynamic flow focusing device used for the shape control synthesis of TTF-Au structures. (B) and (C) SEM images of the structures collected at a FRR of 20 and 0.2, respectively. Adapted from ref. 22 with permission from American Chemical Society, copyright 2014.

In the field of molecular electronics, control over the self-assembly process (or arrangement) of redox active moieties is of paramount importance in harnessing material properties and function.^15^ For example, redox active units such as the tetrathiafulvalene (TTF) have been used (and chemically derivatized) to prepare various conductive systems. A variety of studies have shown that TTF organizations with strong sulfur–sulfur interactions between TTF units and π–π stacked TTF moieties can generate conductive materials when exhibiting a conjugated electronic structure.^16^ TTF is an electron donor and when appropriately organized, charge delocalization can occur *via* doping with an oxidizing agent,^17^ or by interaction with an electron acceptor. Typically, well-conjugated TTF assemblies are obtained in an empirical (or trial-and-error) approach, based on chemical modification of the TTF building blocks.^18^ Recently, Puigmartí-Luis and co-workers studied the self-assembly of TTF under laminar flow conditions within a single layer microfluidic device (Fig. 2A).^19^ Different π–π stacked TTF structures could be generated through the controlled oxidation of a TTF solution with an oxidizing solution containing gold ions (Au^3+^), in acetonitrile. The authors showed that by changing the flow rate ratio (FRR) during synthesis, different TTF-Au structures (with various morphologies) could be selectively generated (Fig. 2B and C). Here, the FRR is defined as the ratio between the total flow rate of the sheath flows and the flow rate of the reagent flows, and determines the degree of hydrodynamic focusing.^20^ When operating under low FRR conditions, a mixture of microcrystalline TTF-Au structures were obtained (Fig. 2B). By increasing the FRR, TTF-Au nanowires could be assembled in a controlled manner (Fig. 2C). Surprisingly, changes in the FRR also led to changes in the π–π stacking distance of the TTF units in the final assemblies (Fig. 2D and E). This study is of special interest for two key reasons. First, the results indicate that hydrodynamic focusing under laminar flow conditions can trigger new assemblies with distinct orientations at the molecular level (*i.e.* a top-down approach is applied to control a bottom-up assembly).^20^ Second, chemical derivatization is not required to modify the molecular arrangement within the collected structures. At this point, it is also worth noting that subtle changes in the π–π stacking distance of TTF assemblies can strongly affect the electrical properties of the final conductive system. Even though the results presented exemplify the benefits of diffusion-limited reactions conducted under hydrodynamic flow focusing conditions,^20^ the variety of TTF-Au structures produced (from triangular to square to round shapes) could not be easily controlled within the microfluidic device employed. The main reason for this is attributed to the no-slip boundary condition present in single layer microfluidic devices (*i.e.* a zero fluid velocity at the liquid/solid-wall boundary), which results in a parabolic velocity profile across the channel.^21^ Accordingly, shape-controlled syntheses of TTF-Au structures could be realised using three-dimensional hydrodynamic flow focusing devices, which can remove the no-slip boundary condition during the reaction of reagent-laden flows. In this regard, the microfluidic synthesis of TTF-Au assemblies in a 3D hydrodynamic focusing device was recently reported.^22^ In this study, the authors showed that Dean vortices generated in curved channels allow for the vertical focusing of TTF and HAuCl_4_ reagent streams (Fig. 3A). By changing the FRR, the formation of different TTF-Au structures with defined morphologies, including branched assemblies, hexagonal structures, dendritic nanostructures, and/or nanowires, could be obtained (Fig. 3B and C). The FRR is defined as the ratio between the total flow rate of the reagent-laden flows and that of the sheath flows. However, it should be noted that in the described microfluidic approach only one of the reagent streams is compressed into the centre of the channel, with the flow of the second reagent enveloping it, while the sheath flows laterally flank the reaction zone (Fig. 3A). Accordingly, it is expected that further optimization of the formation of π–π stacked TTF structures can be successfully achieved with microfluidic systems that comprise 3D channel geometries in which the reaction zone is completely surrounded by the sheath flow.

#### Inorganic and organic materials

**Fig. 4:**
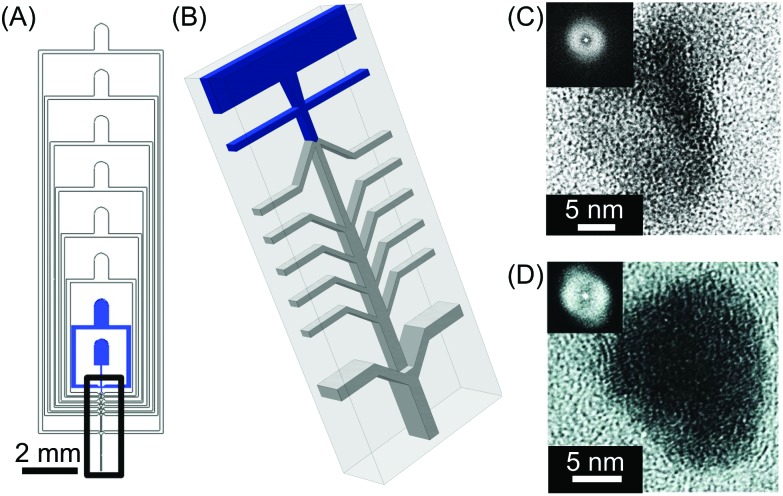
(A) General view of the microfluidic nebulator used. (B) Zoom-in illustration of the 3D channel geometry present in the microfluidic device used in this study. The blue inlets indicate the microfluidic channels where the liquids are injected inside the device while only air is introduced in the other inlets. (C) and (D) High-resolution transmission electron microscope (HRTEM) images of CaCO_3_ and fenofibrate nanoparticles produced with this microfluidic method, respectively. The insets in (C) and (D) are the Fourier transform of the HRTEM micrographs presented, indicating the amorphous nature of the materials generated. Adapted from ref. 23 with permission from The American Association for the Advancement of Science, copyright 2015.

Amstad and co-workers have recently shown that microfluidic devices with 3D channel geometries can be used to produce amorphous nanoparticles of inorganic and organic materials that usually have a high tendency to crystallize, *e.g.* CaCO_3_, NaCl, or fenofibrate (a generic organic drug).^23^ In this work, a supersonic air flow was used to produce small droplets of solutions (approximately 100 nm in diameter) that dry on timescales faster than crystal nuclei formation. Such fast evaporation enables the formation of kinetically trapped amorphous states of the materials under investigation, without crystallization inhibitors. The 3D microfluidic device used in this study consisted of two reagent-laden flows that mix prior to droplet formation, and six pairs of air inlets (Fig. 4A). The sixth air inlet pair intersects the nebulized reagent-laden flow in a microfluidic channel of larger cross-sectional dimensions, which results in a 3D channel geometry (Fig. 4B). Furthermore, the authors demonstrated that the method can enable the scaled-up production of amorphous nanoparticles by operating three microfluidic devices in parallel, with a throughput of 15 mg hour^–1^ of material. These results provide compelling evidence that microfluidic methods can lead to the control of self-assembly processes such as crystallization. The ability to control crystallization is crucial in many technological fields. For example, amorphous structures can have different physicochemical properties compared to their crystalline counterparts, such as increased solubility. This can be of considerable help for drug bioavailability.

Control of crystallization processes has also been reported through the use of liquid–liquid interfacial reactions. The reaction and diffusion-limited conditions offered by hydrodynamic flow focusing formats can induce (and indeed control) concentration gradients, which in turn may result in kinetic control over crystallization. It should also be noted that crystallization is a self-assembly process, where the diffusion of molecules and the way they interact are key factors for its control and adjustment. In this regard, the design of novel architectures for microfluidic devices (*e.g.* for 3D hydrodynamic focusing) can result in an improved control over the reaction diffusion zone, giving even more possibilities for tuning materials synthesis. In this context, computational simulations of fluid dynamics (analysis of fluid forces, diffusion of reactants, concentration gradients) are becoming popular tools, which can greatly help in the validation and optimization of the microfluidic designs, as well as in the mechanistic elucidation of self-assembly processes.^22^

#### Coordination polymers (CPs)

**Fig. 5:**
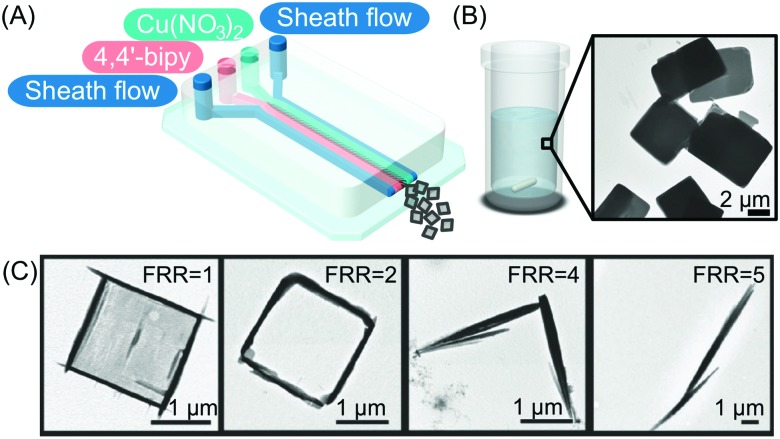
(A) Illustration of the microfluidic setup used in the experiments. (B and C) Transmission electron microscope (TEM) images of the CP synthesized in flask mixing experiments and with the continuous-flow microfluidic device at different FRRs, respectively. Adapted from ref. 24 with permission from Wiley-VCH Verlag GmbH & Co., copyright 2016.

Rubio-Martinez and co-workers recently showed that controlled RD conditions encountered in continuous-flow microfluidic devices can be used to control, isolate and study out-of-equilibrium crystal structures.^24^ In this study, the authors isolated kinetically trapped crystal states of a coordination polymer made through the reaction of 4,4′-bipyridine and Cu(NO_3_)_2_·6H_2_O. CPs formed *via* non-covalent metal/organic ligand interactions are the focus of extensive research due to their potential applications in catalysis, sensing, and molecular separation. Accordingly, technologies that can isolate non-equilibrium states of CPs and shed light on their mechanisms of self-assembly are of high priority. As shown in Fig. 5A, the microfluidic device used in this study consisted of four input channels and one outlet. Two side channels (sheath flows) were used to modulate the reaction zone volume and concentration gradients of injected reagent solutions. The results presented indicated that while square plate-like crystals are always produced in flask mixing experiments (Fig. 5B), partially filled frames, hollow frames, truncated hollow frames, and needles can be selectively isolated by increasing the FRR in microfluidic experiments (Fig. 5C). Indeed, XRPD data indicated that all crystal morphologies generated under microfluidic-guided assembly are structurally identical, and in addition identical to XRPD patterns measured for CPs prepared through flask mixing. This subsequently prompted the authors to consider the crystals produced by microfluidic mixing as non-equilibrium forms isolated along the self-assembly (crystallization) pathway. To conclude, this study provided compelling evidence that diffusion-limited microfluidic environments can provide valuable insights into crystallization processes. Indeed, the ability to study kinetically trapped states and non-equilibrium intermediates can ultimately lead to new assemblies with unique properties.

#### Covalent organic framework (COF)

**Fig. 6:**
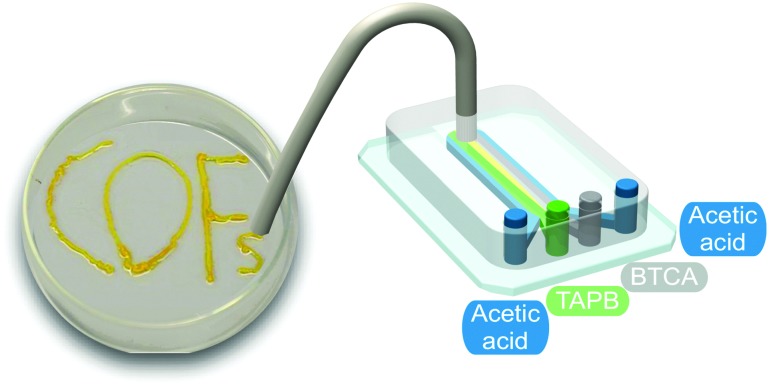
Illustration of the microfluidic device used for the synthesis and printing of the COF. Adapted from ref. 25 with permission from The Royal Society of Chemistry.

In the previous discussion, microfluidic devices were successfully used to isolate non-equilibrium self-assembled structures. In addition, kinetically trapped or metastable states^14^ can be efficiently avoided through the use of microfluidic-based processing methods. This idea was recently exploited in the microfluidic synthesis of a covalent organic framework (COF).^25^ COFs are crystalline porous materials generated by the integration of organic molecules *via* covalent bonds. In this work, the synthesis of an imine based COF material by the condensation of 1,3,5-tris(4-aminophenyl)benzene (TAPB) and 1,3,5-benzenetricarbaldehyde (BTCA) in acetic acid was reported (Fig. 6). The imine bond can form and break rapidly under acidic conditions, enabling the material generated inside the chip to reach thermodynamic equilibrium. The results presented demonstrate that a microfluidic approach can produce a highly crystalline and porous COF material consisting of fibrillary microstructures. In flask-based experiments, only sub-micron particles of this COF material could be obtained.^26^ Furthermore, the extraordinary mechanical stability of the COF fibers eluted from the microfluidic device allowed the direct drawing of the COF over different surfaces (Fig. 6), making possible the printing of 2D and 3D COF structures.

#### Metal–organic frameworks (MOFs)

**Fig. 7:**
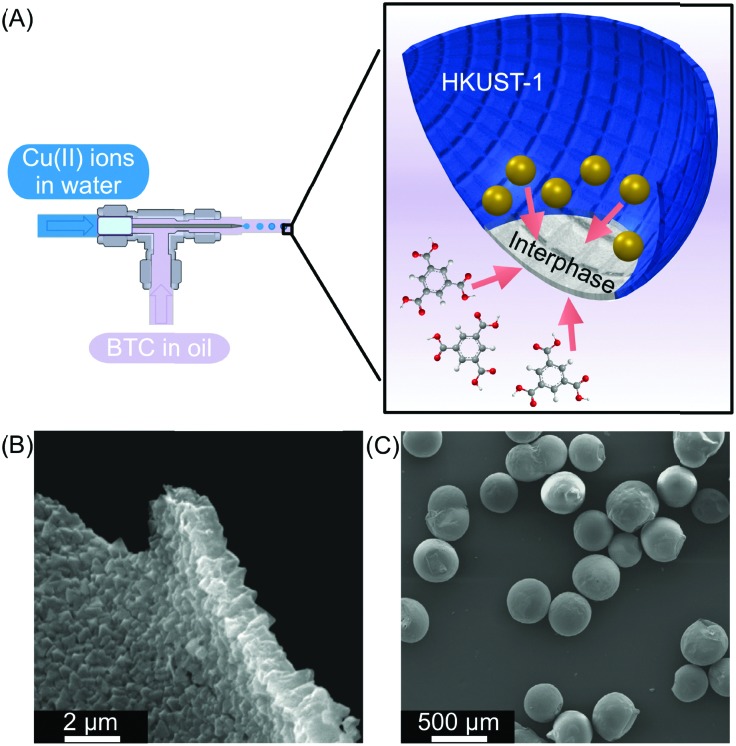
(A) Microfluidic setup used to create water in oil droplets. The oil phase contains the BTC ligand and the water phase contains Cu(ii) ions. At the interphase, a HKUST-1 film is generated (blue). (B) and (C) SEM images showing the homogeneity of the HKUST-1 shell and the capsules, respectively. Adapted from ref. 27 with permission from Springer Nature, copyright 2011.

Liquid–liquid interfacial reactions have also been used to control the self-assembly of porous crystalline CPs, also referred as metal–organic frameworks (MOFs). For example, Ameloot and co-workers used a droplet-based microfluidic approach to generate water droplets (containing a metal ion) in an immiscible continuous oil phase containing an organic ligand (Fig. 7A).^27^ The liquid–liquid interface generated between these two immiscible solvents was then used as a template to synthesize MOF layers. The authors proved the synthesis of HKUST-1 (a prototypical MOF) through the reaction of Cu(ii) ions with 1,3,5-benzenetricarboxylate (BTC) ligands. Interestingly, the diffusion of Cu(ii) ions (dissolved in water) and BTC (dissolved in the oil phase) towards the water–oil interface assisted the formation of defect-free MOF layers that have a controlled thickness and excellent homogeneity (Fig. 7B). The results presented in this seminal contribution are in sharp contrast to other synthetic approaches where MOF films are fabricated in an uneven and discontinuous manner.^28^ Moreover, the MOF structures created inside the microfluidic device were characterized as hollow crystalline capsules (Fig. 7C), which confirms the templating role of the droplet in MOF self-assembly. This result is of high importance since compact and regular MOF films are expected to exploit their tuneable porosities in a broad range of applications, such as gas storage and separation, catalysis, or purification.

The confined synthesis of MOFs inside droplets has also been reported by Kim and co-workers.^29^ In this study, multiple MOF materials were synthesized, where both metal ions and organic ligands were dissolved in the discontinuous phase. In this scenario, the continuous oil phase is only used for droplet generation. The reduction of the reaction volume into droplets proved to be a crucial factor for the ultrafast synthesis of different and high-quality MOF crystals, such as HKUST-1, MOF-5, IRMOF-3, and UiO-66.^29^ In comparison to flask-based synthetic approaches, the representative MOF crystals prepared could be assembled within a few minutes under solvothermal conditions, whilst several hours or days are required when using conventional flask-based techniques. The large surface area-to-volume ratio inside such droplets and excellent heat and mass transfer facilitated the formation of high-quality MOF crystals in a time-efficient manner (see Table 1). Finally, the authors were able to confirm that two distinct consecutive synthetic steps can be integrated in their microfluidic method, resulting in the formation of different types of core–shell MOF composites within minutes. These results highlight the superiority of microfluidic methods, not only in accelerating the kinetics of self-assembly processes, but also in the judicious design of complex multifunctional structures. For example, the authors showed the rationalized and continuous synthesis of core–shell MOF composites where each MOF can have a property but the whole structure can lead to a new function.

### Surface-based syntheses

Microfluidic technologies also represent a valuable tool for the controlled growth, manipulation and processing of self-assembled materials at surfaces (Fig. 1), a task which is crucial for their eventual integration into functional devices.

#### MOFs

**Fig. 8:**
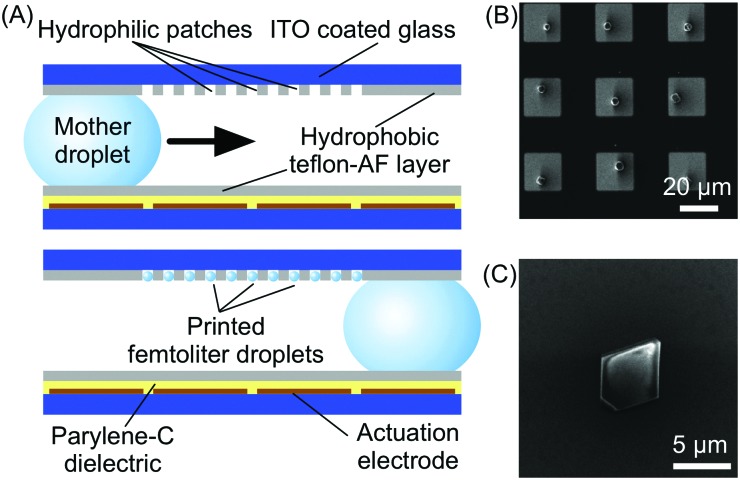
(A) Schematic illustration of the digital microfluidic device used in this experiment and of its functioning. (B) SEM image showing the array of HKUST-1 crystals fabricated by this technology. (C) Details of a single HKUST-1 crystal. Adapted from ref. 30 with permission from Wiley-VCH Verlag GmbH & Co., copyright 2012.

Witters and co-workers recently reported an accurate and high-throughput integration method of HKUST-1 MOF crystals onto surfaces employing a digital microfluidic approach.^30^ Digital microfluidic devices allow the manipulation of discrete droplets on surfaces through electrostatic actuation. In brief, using this approach, the contact angle of the droplet with respect to the surface holding it can be effectively modified through application of an electric field. This change of contact angle facilitates the controlled movement of the droplet on the surface. In this study, the authors used a modular two-plate microfluidic device in which the bottom plate incorporated the electrode array (used to control droplet trajectories) and the top plate contained a network of hydrophilic micro-patches patterned in a hydrophobic surface (Fig. 8A). The authors demonstrated that when a polar droplet containing MOF precursors is transported over the hydrophilic pattern, smaller (femtoliter) droplets can be generated inside the hydrophilic patches due to their chemical affinity with the surface (Fig. 8A). By controlling the evaporation of the small droplets, single crystals of HKUST-1 could be generated at defined positions inside the hydrophilic micro-patches. Additionally, the authors demonstrated that the approach could also be used to tune the size of HKUST-1 single crystals by modifying the dimensions of the hydrophilic micro-patches. The results indicate that microfluidic-based methods are able to accurately deposit single crystals onto a surface, and are also an ideal technology for performing parallelized self-assembly of materials of high monodispersity.

#### CPs

**Fig. 9:**
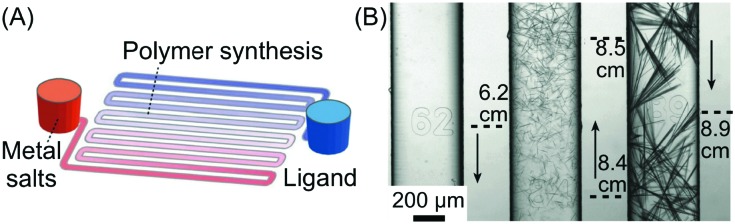
(A) Schematic illustration of the device used for the synthesis of silver acetylides. (B) Microphotographs showing the distribution and morphology of silver acetylide crystals formed along the microchannel. Adapted from ref. 31 with permission from Wiley-VCH Verlag GmbH & Co., copyright 2015.

The high surface area-to-volume ratio present in single layer microfluidic devices (Table 1) has also been used to excellent effect in self-assembling materials that are not or not easily obtained in flask-based mixing experiments. As a representative example, Cao and co-workers reported the self-assembly of three CPs (silver acetylides), as single-crystals, *via* long-term counter diffusion of reactant solutions inside a microfluidic channel (Fig. 9A).^31^ Indeed, until this time, the formation of high-quality single crystals of silver acetylides was a challenge for the crystallographic community. Additionally, by capitalizing on the ability to control reagent diffusion inside microfluidic channels and knowledge of the diffusion coefficients of the reagents, the authors demonstrated the controlled and rationalized self-assembly of silver acetylide crystals at specific positions upon a planar surface (Fig. 9B). Using such microfluidic tools, it is expected that microfluidic devices will provide valuable information relating to the coordination principles (and/or interactions) of molecular-based materials that are inaccessible using traditional macroscale methods, and thus may provide a novel route to achieve unique structures and properties.

#### Organic-based conductors

**Fig. 10:**
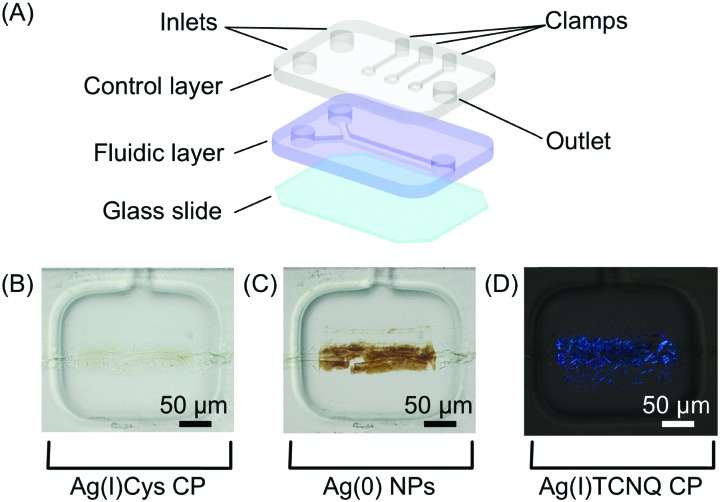
(A) Schematic view of the two-layer microfluidic platform employed in the study. (B–D) A sequence of optical microscope images showing the Ag(i)Cys CP assembled at the reactant interface; its reduction to Ag(0); and the subsequent formation of Ag(i)TCNQ (cross polarized image), respectively. Adapted from ref. 32 with permission from American Chemical Society, copyright 2014.

Even though the above studies demonstrate the accurate localization of self-assembled materials on a surface, the direct integration of deposited materials with read-out components is required for device fabrication and operation. To address this issue, Puigmartí-Luis and co-workers demonstrated spatial control in the synthesis and integration of a representative metal–organic semiconductor complex onto an electrode array patterned on a planar surface.^32^ Specifically, the authors reported the formation of silver-tetracyanoquinodimethane (Ag(i)TCNQ), using a two-layer microfluidic platform (structured in PDMS) that incorporates pneumatic clamps (Fig. 10A). The control layer (top) contains square shaped deformable clamps that can be actuated, through the application of nitrogen gas pressure, to trap structures synthesized in the fluidic channel below. Employing this novel device, Ag(i)TCNQ was synthesized in a stepwise manner, employing consecutive chemical reactions upon self-assembled structures trapped on the surface. Briefly, a silver-cysteine (AgCys) CP was initially formed in the fluidic layer by injecting AgNO_3_ and Cys solutions through two inlet channels. Bundles of AgCys nanowires were locally trapped on a glass substrate by actuating a clamp (with an applied pressure of 3 bar), followed by removal of surplus reagents with a flow of water (Fig. 10B). In a second step, the clamp was partially released (reduced to 1 bar), and a solution of ascorbic acid was injected in the microfluidic channel to reduce the Ag(i) present in the trapped AgCys CP to silver metal, Ag(0) (Fig. 10C). Finally, after a washing step, a saturated solution of TCNQ was injected, leading to the formation of Ag(i)TCNQ (Fig. 10D). In this reaction, Ag(0) acts as a nucleation site for the formation of the metal–organic semiconductor complex (*i.e.* Ag(i)TCNQ). Additionally, the authors employed microfluidic control to integrate Ag(i)TCNQ nanowires onto electrode arrays patterned on the glass surface, and subsequently measured their electrical properties. In general, this study demonstrated the significant advantage of microfluidics for the localized synthesis of functional materials on surfaces and their direct integration into devices. Critically, the adoption of microfluidic tools avoids the need for multi-step processing protocols that are likely to compromise the integrity of functional assemblies, once they are generated.

**Fig. 11:**
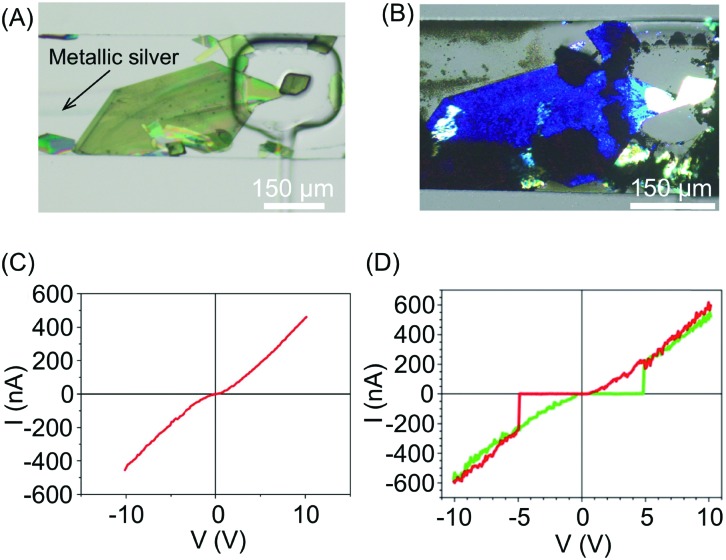
(A) Optical image of a large TCNQ crystal grown in the fluidic channel and trapped with a pneumatic clamp, and a line of generated silver metal. (B) Polarized optical image of the Ag(i) doped TCNQ crystal. The white areas correspond to the pure TCNQ, while the rest of the crystal shown is the Ag-TCNQ complex. (C) *I*/*V* sweep of the Ag(i)TCNQ complex and (D) of the intermediate region measured with CAFM. Adapted from ref. 33 with permission from The Royal Society of Chemistry.

In a continuation of this work, the authors subsequently constructed molecular circuits from a TCNQ crystal.^33^ Specifically, a microscopic discrete crystal of TCNQ was used as a template for the *in situ* synthesis of Ag(i)TCNQ. To this end, the authors used the same two-layer microfluidic chip originally described. First, a TCNQ crystal was grown and immobilized under a clamp. This was then reacted with Ag(0) generated in the fluidic channel by the chemical reduction of Ag(i) (Fig. 11A). The different materials generated could be distinguished under polarized light, with unreacted TCNQ under the clamp appearing as bright white, while the segments of the crystal that reacted with Ag(0) (*i.e.* forming the Ag(i)TCNQ complex) appearing as different shades of blue, indicating a significant degree of structural order (Fig. 11B). The crystal shown in Fig. 11B was analysed off-chip by atomic force microscopy (AFM), revealing three regions possessing different electric properties: one insulating region corresponds to pure TCNQ; a conductive region represents the full conversion of TCNQ into the semiconductor Ag(i)TCNQ complex (Fig. 11C); and an unprecedented state found between the insulating and conductive areas that exhibits bipolar resistive switching (Fig. 11D).

Electrochemical processes carried out in microfluidic devices have also been used to accomplish controlled self-assembly and direct integration of functional materials in electrode arrays located on a surface. For example, Tseng and co-workers showed that hydrodynamic focusing,^20^ present in single layer microfluidic devices (Table 1), can be successfully used to induce site-specific growth of polypyrrole (Ppy), a conductive organic polymer.^34^ Ppy fibres with different widths (on the micron scale) could be generated by varying the focusing conditions of the precursor pyrrole solution. Furthermore, the authors showed that these structures could be directly used as sensor elements. Herein, and in contrast to all previous examples, an external input (current) was employed to trigger the self-assembly and growth of the investigated material (Ppy in this case) at defined positions on the device surface.

#### Inorganic materials

**Fig. 12:**
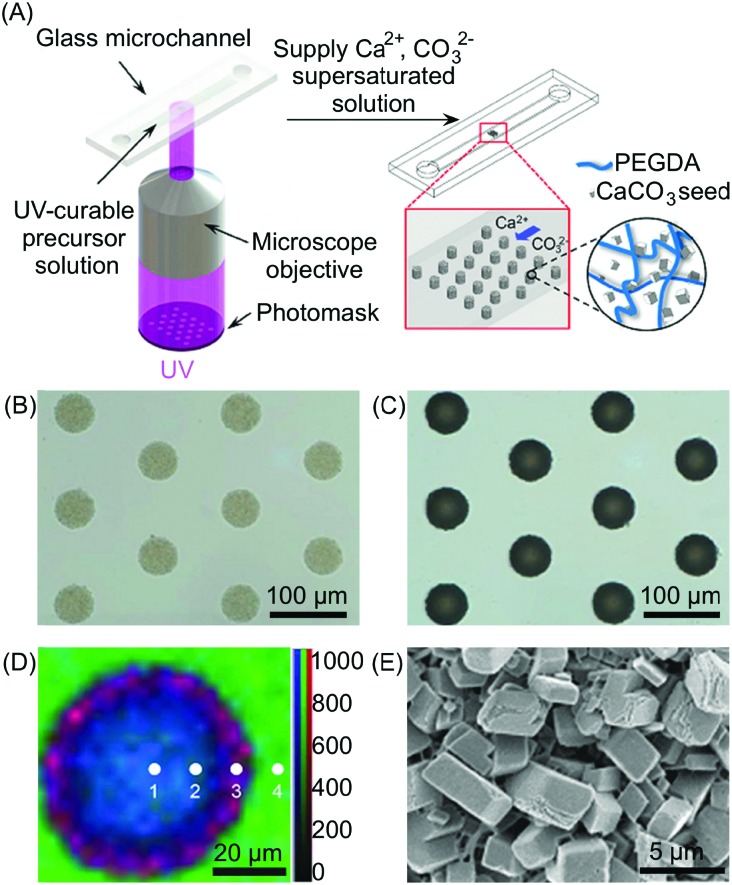
(A) Schematic illustration of the setup used for the photolithographic patterning and the subsequent *in situ* growth of the CaCO_3_. (B and C) Optical microscopy images of CaCO_3_/polymer composite microstructures before (B) and after (C) growth of the CaCO_3_. (D) Raman mapping of the composite post of (C) with different positions (1–4) obtained by integrating over the wavenumber ranges of CaCO_3_ (1050–1150 cm^–1^, red), organic (2800–3000 cm^–1^, blue), and water (3000–3700 cm^–1^, green), respectively. Mapping data were normalized to the strongest intensity of the CaCO_3_. (E) SEM image of the outer side surface of the composite posts. Adapted from ref. 35 with permission from Wiley-VCH Verlag GmbH & Co., copyright 2016.

Another interesting example is the use of UV radiation as an external trigger to site-selectively self-assemble materials onto surfaces of microfluidic devices. Indeed, optical methods such as microscope-based photolithography have proved to be powerful in controlling not only the localization of the self-assembled materials on surfaces, but their growth, aspect ratio, and physical properties. Microscope-based photolithography is a micropatterning technique that involves focusing a projected image (generated using a photomask) onto a photocurable monomer that only polymerizes in the exposed regions. This approach can also be used in microfluidic devices (Fig. 12A), hence providing a high-throughput method for the generation of photo-crosslinkable structures on microfluidic surfaces. For example, Doyle and co-workers have recently demonstrated the potential of this approach through the site-selective growth of CaCO_3_ by photopatterning composite microstructures on a microfluidic channel.^35^ The composite microstructures reported consist of poly(ethylene glycol) diacrylate (PEGDA), used as a photocurable monomer, and CaCO_3_ nanoparticles, employed as seed crystals for CaCO_3_ growth. The authors showed that by supplying supersaturated solutions of Ca^2+^ or CO_3_^2–^ ions into the microfluidic channel, selective mineralization on the composite microstructures can be achieved due to the enhanced mass transfer offered by the microfluidic environment (Table 1). This novel method also enabled an unprecedented control over the physical properties of CaCO_3_, with well-defined crystal facets and smooth surface morphologies being demonstrated. These results can be attributed to local differences in the concentration of the reagent solutions in the microfluidic channel, which can be precisely controlled.

## 3. Supramolecular chemistry and microfluidic technologies

Supramolecular chemistry typically deals with self-assembled structures at thermodynamic equilibrium, *i.e.* structures residing at the global minimum of a free energy landscape.^14^ In such systems, the internal structure and morphology of the formed assemblies only depend on the information ‘encoded’ in the molecular components, and on the final experimental conditions (such as concentrations, solvent composition, temperature), and are independent of the exact path by which monomers self-assemble. Recently, however, there has been a growing interest in non-equilibrium supramolecular systems, which are either kinetically trapped (*i.e.* located at local energy minima) or dissipative (*i.e.* requiring a continuous influx of energy/chemical inputs to form and survive).^14^ Under non-equilibrium conditions, the kinetics of the self-assembly play a critical role, and may influence the final outcome of the process. Different preparation protocols have been explored to regulate competitive aggregation pathways, which may lead to different structural assemblies.^14^

Usually, for example, in flask-based synthesis, self-assembly is triggered by the addition of a poor solvent, or the application of a chemical stimulus (such as the addition of protons, metal ions or co-assembling species) to a solution of building blocks, followed by manual agitation or vortex stirring. The most common approximation assumes that all molecules are exposed to the same (homogeneous) chemical environment, so that the aggregation will proceed randomly in space and time. On the contrary, the omnipresent local gradients in reactant concentrations (and solvent composition), caused by imperfect mixing and extended diffusion times, are almost always ignored.^14^ While the latter does not affect the outcome in the case of equilibrium self-assembly, the presence of a non-uniform chemical environment is likely to strongly influence the structure of the aggregates formed under kinetic control. This happens, for example, when molecular diffusion and nucleated growth occur on similar timescales, and/or when strong non-covalent interactions (hampering equilibration) are involved. In this context, and as we will show, precise spatio-temporal control over reactant (or solvent) diffusion, enabled by microfluidic technologies, turns out to be a powerful tool in selecting the desired aggregation pathway(s), ultimately leading to aggregates with different tailored features (structure and shape). Additionally, we will show how microfluidic technologies are invaluable in the mechanistic investigation of supramolecular self-assembly processes, since they are able to extract information that is otherwise inaccessible, such as the presence of metastable states, the role of reactant concentration gradients and of fluid velocities in aggregation, as well as the direct visualization of the growth process down to the level of a single assembly.

### Supramolecular chemistry in flow

As discussed in the previous sections, the peculiar hydrodynamic properties of solutions flowing in microfluidic channels (in particular the existence of laminar flow and lack of turbulence)^20^ allow the precise control of reagent concentration gradients and solvent composition in both space and time.^9^ In addition, the possibility of modulating multiple parameters, such as flow rates, flow focusing and channel geometries, allows the fine-tuning of the RD zone in a top-down manner. Recently, with these ideas in mind, a number of research groups have used continuous flow microfluidic platforms for the controlled preparation of non-equilibrium supramolecular polymers from different building blocks.

**Fig. 13:**
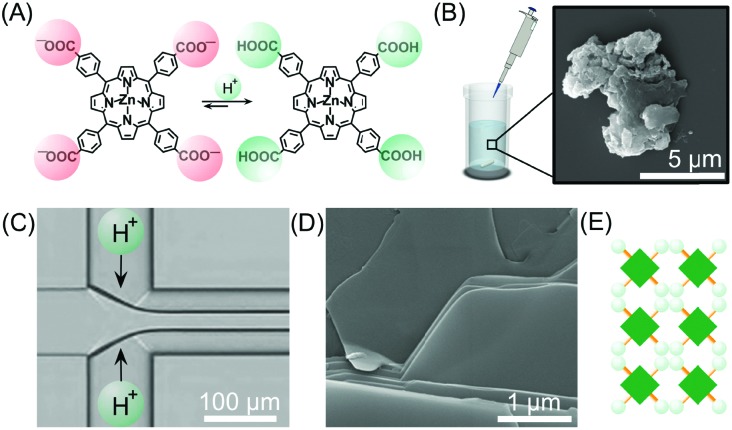
(A) Chemical structure of ZnTCPP in the anionic and protonated forms. (B) Exemplary SEM image of the amorphous aggregates formed under flask mixing. (C) Schematic illustration of the setup used in this experiment. (D) SEM image of the multi-layered flakes formed by microfluidic mixing. (E) Schematic representation of the 2D hydrogen-bonded network formed under kinetic control (fast protonation). Adapted from ref. 36 with permission from The Chemical Society of Japan, copyright 2015.

**Fig. 14:**
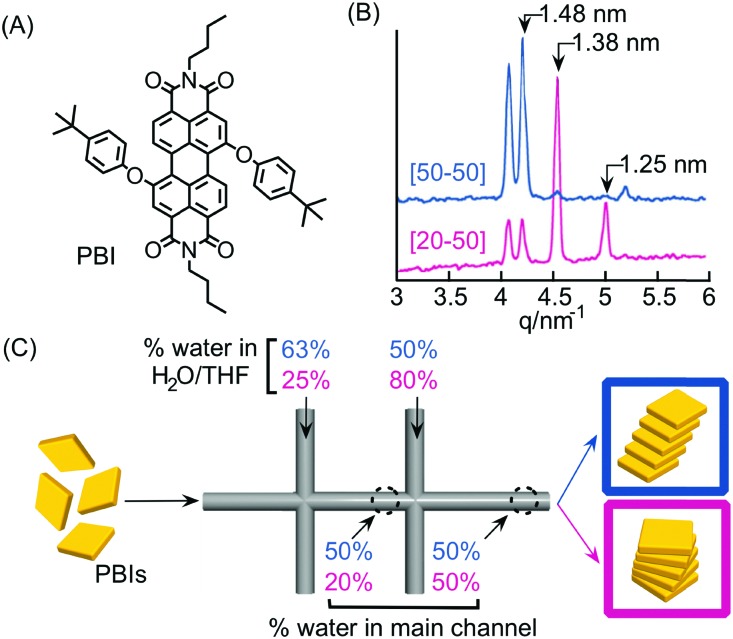
(A) Chemical structure of PBI. (B) Synchrotron XRD patterns of the PBI assemblies formed under different flow conditions, *i.e.* different solvent compositions at the first cross-point, reported in (C). (C) Schematic representation of the double cross point microfluidic setup used. Blue and pink sets of numbers correspond to different experiments yielding respectively H- and J-aggregates. Note that the solvent composition at the outlet is the same for both the experiments. Adapted from ref. 37 with permission from The Chemical Society of Japan, copyright 2015.

For example, Numata and co-workers exploited controlled proton diffusion achieved in a hydrodynamic flow focusing device^20^ for the formation of kinetically stable porphyrin assemblies based on 2D hydrogen-bonded networks (Fig. 13).^36^ When a basic solution of the tetracarboxylate porphyrin ZnTCPP (Fig. 13A) was injected in the central stream of a cross-channel microfluidic device, and squeezed between two lateral streams of aqueous HCl (Fig. 13C), micron-sized flakes with a multi-layered structure were formed (Fig. 13D). On the other hand, only amorphous assemblies were observed upon flask mixing with the same final conditions of concentration and pH (pH < 4) (Fig. 13B). Off-chip X-ray diffraction analysis of the structures generated inside the microfluidic device suggested a molecular packing based on a regular cyclic H-bonding moiety between four adjacent monomers (Fig. 13E). The formation of extended multi-layered flakes under microfluidic mixing can be ascribed to the cooperative effect of synchronous protonation of a large number of –COOH groups (rapid proton diffusion in the thin porphyrin-laden stream), with the ‘templating’ effect of the liquid–liquid interface (*i.e.* orientation of clusters under laminar flow), which favour the creation of regularly extended 2D networks. Remarkably, these non-equilibrium 2D porphyrin assemblies were found to be very robust and only decomposed over 24 hours in a vial at neutral pH (due to slow deprotonation of –COOH groups). On the other hand, when a solution of the microflakes was re-injected in the central channel and squeezed by two alkaline solutions, so as to have the same final neutral pH, the flakes disassembled within milliseconds. Here, the local high concentration of deprotonated –COOH groups achieved inside the microfluidic device leads to a sudden electrostatic repulsion that destabilizes the 2D porphyrin aggregates. This result confirms the utility of microfluidic mixing in overcoming the kinetic barrier along the path leading to the thermodynamic state (*i.e.* monomeric porphyrins at neutral pH). In another example, the precise spatial control of poor/good solvent gradients across and along the channel has been exploited to modulate the supramolecular structure of perylene bisimide (PBI) aggregates formed by π–π stacking and hydrophobic interactions (Fig. 14).^37^ It should be noted that the possibility of mastering such low-directional, non-covalent interactions in a top-down manner is a worthwhile challenge in supramolecular chemistry. In this example, a PBI solution in THF (a good solvent) was injected into the central channel of a double cross device, and squeezed between water (a poor solvent) or THF/water streams at the first cross point, whilst the final solvent composition was adjusted by injection of an appropriate THF/water mixture at the second cross point (Fig. 14C). The distance between the two crossing points was fixed at 10 mm. The nucleation rate and self-assembly dynamics of PBI molecules could be modulated by changing the polarity of the side sheath flows and/or the width of the central PBI-laden stream (*i.e.* the hydrodynamic focusing affecting the rate of water diffusing across the PBI-laden stream), resulting in different molecular packing of PBI aggregates (Fig. 14C). Specifically, H-like aggregates were selectively formed when a higher water content was used at the first cross point, corresponding to higher aggregation rates (*e.g.* faster water diffusion yields a higher number of PBI molecules in contact with the poor solvent; blue labelling in Fig. 14C), whereas J-like aggregates were the predominant species at lower aggregation rates (lower solvent polarity at the first cross point; pink labelling in Fig. 14C) as evidenced by XRD analysis (Fig. 14B). The latter is a remarkable example of pathway selection in which solvent diffusion is on the same timescale of nucleation and growth.

**Fig. 15:**
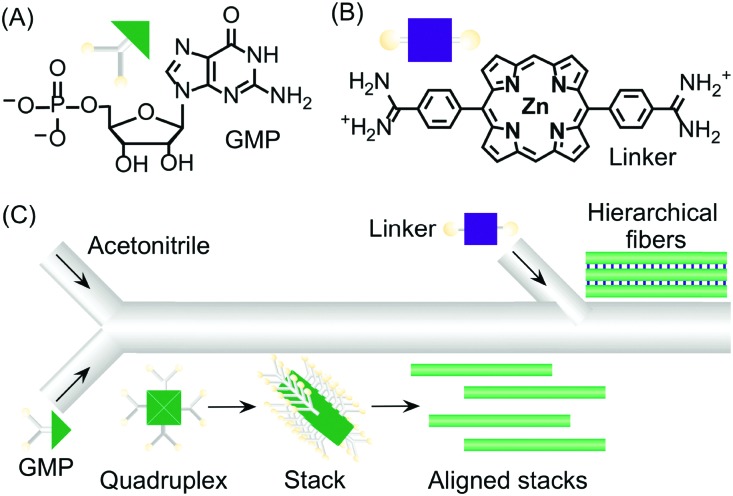
(A) Chemical structure of GMP. (B) Chemical structure of the bis-cationic linker porphyrin. (C) Schematic illustration of the microfluidic setup used in the experiment and of the hierarchical growth of GMP microfibers by stepwise activation of non-covalent intermolecular interactions by spatial and temporal control. Adapted from ref. 38 with permission from Wiley-VCH Verlag GmbH & Co., copyright 2013.

Continuous flow devices have also been used as unique tools in enabling different intermolecular interactions at specific points in space and time along a microfluidic channel, which in turn allows the controlled growth of hierarchical supramolecular structures. As an example, the formation of kinetically trapped hierarchical fibers of guanosine 5′-monophosphate (GMP) was achieved by stepwise activation of hydrogen bonding (and π–π stacking), followed by electrostatic salt bridging along a microfluidic channel (Fig. 15).^38^ When acetonitrile (a poor solvent) was gradually diffused into an aqueous solution of GMP under continuous flow, the formation of H-bonded GMP quadruplexes, followed by their stacking into nanofibers, occurred. The GMP anionic nanofibers created after the first junction tend to align along the microfluidic channel due to the hydrodynamic effects of the laminar flow (Fig. 15C). The aligned stacks can be regarded as a metastable configuration of the supramolecular system that only survives under continuous flow (*i.e.* non-equilibrium conditions). When a bis-cationic linker was injected from a second inlet along the microfluidic channel, this supramolecular arrangement was frozen (kinetically trapped) leading to the formation of micron-sized fibrous aggregates made by the regular alignment of cross-linked GMP nanofibers (Fig. 15C). Remarkably, when aqueous GMP was mixed with acetonitrile in a vial at the same concentration and solvent conditions, aggregation to nanofibers was not observed. Similarly, when eluted nanofibers were mixed with the cationic linker off-chip, no bundled micro-structures were formed, thus demonstrating once more the strong role of microfluidic mixing in enhancing self-assembly and its potential in controlling the hierarchical formation of supramolecular structures from multiple molecular components.

**Fig. 16:**
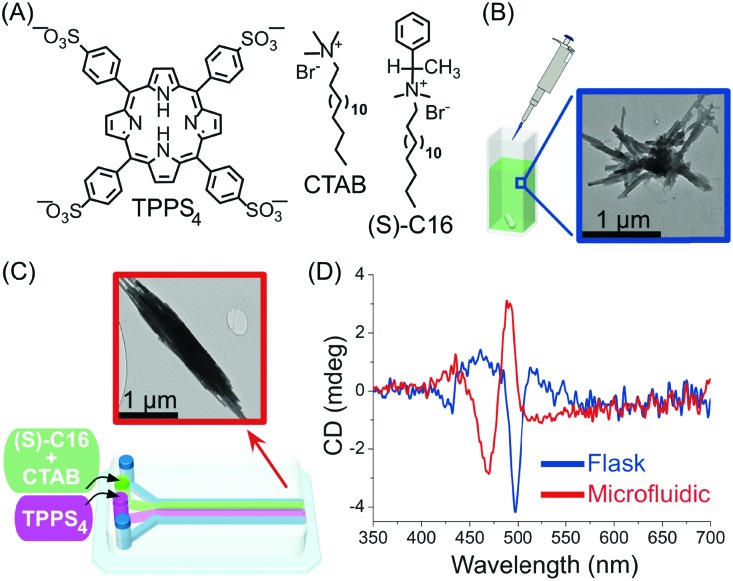
(A) Chemical structure of H_2_TPPS_4_, CTAB and of the chiral amphiphile (*S*)-C16. (B) TEM image of exemplary aggregates formed by flask synthesis. (C) Schematic illustration of the microfluidic setup used in this example and representative TEM image of the well-aligned rod-like aggregates formed under microfluidic mixing. (D) Circular dichroism spectra showing the effect of microfluidic *versus* flask mixing on the chirality of the formed H_2_TPPS_4_/CTAB heteroaggregates, *i.e.* on the efficiency of the chiral induction. Adapted from ref. 39 with permission from American Chemical Society, copyright 2016.

In another example, Sorrenti and co-workers showed how the diffusion-controlled mixing of reactants at very early stages within the self-assembly process – as enabled by continuous-flow microfluidic devices – can significantly affect the induction of supramolecular chirality in a multicomponent system.^39^ Specifically, the authors investigated the heteroaggregation of H_2_TPPS_4_ porphyrin with cetyltrimethylammonium bromide (CTAB) in the presence of small amounts of the chiral amphiphile (*S*)-C16, as a chiral inducer (the ‘‘sergeant’’), under both microfluidic (laminar) and flask conditions (Fig. 16). While the flask synthesis produced metastable assemblies whose supramolecular chirality slowly evolved with time (over 3 days) to those observed in the absence of the inducer, microfluidic mixing allowed for efficient chiral induction, even at low concentrations of the inducer, as shown by circular dichroism (Fig. 16D). Furthermore, different morphologies of the aggregates prepared by each kind of mixing were observed (Fig. 16B and C), with the microfluidic samples showing clusters of well aligned rod-like aggregates (Fig. 16C). Remarkably, only 20 milliseconds of laminar flow conditions at the nucleation stage of the self-assembly process were enough to control the complex behaviour underlying transcription of supramolecular chirality in the multicomponent system studied, and resulting in an enhanced effect of the chiral inducer in the microfluidic mixing experiments.

**Fig. 17:**
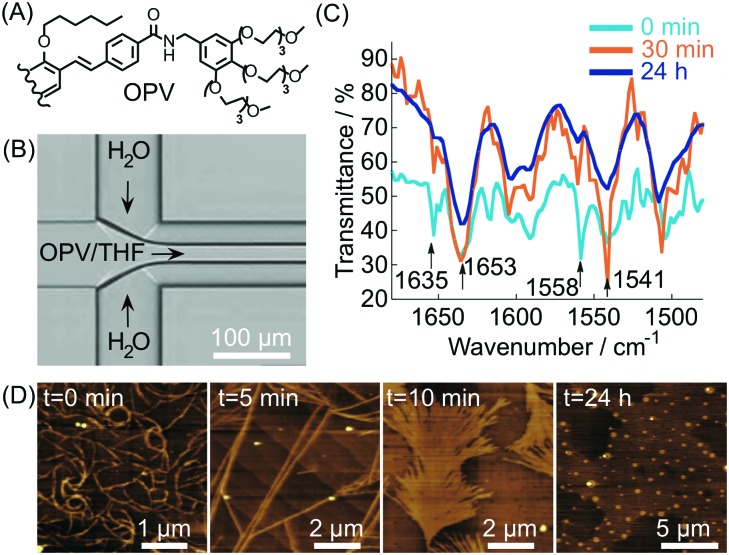
(A) Chemical structure of OPV (half molecule is shown for clarity). (B) Schematic illustration of the microfluidic setup used in the experiment. (C) Time dependent IR spectra of the samples prepared by microfluidic mixing. (D) AFM images showing the morphological change with time of the assemblies formed under microfluidic mixing. Adapted from ref. 40 with permission from The Chemical Society of Japan, copyright 2015.

In most of the examples presented so far, the cooperative formation of strong non-covalent interactions (enhanced under microfluidic conditions) is responsible for the high kinetic stability of the formed aggregates. Conversely, microfluidic mixing can be used to choose the self-assembly pathway leading to metastable non-equilibrium systems,^14^ either surviving only under flow within the chip or for a short time after elution. For example, in this scenario, it has been reported^40^ that the self-assembly of an oligo(*p*-phenylenevinylene) based amphiphile (OPV) (Fig. 17A), triggered by controlled diffusion of water (a poor solvent) in a cross point-microfluidic setup (Fig. 17B), leads to metastable flexible fibers primarily stabilized by π–π stacking interactions. However, such aggregates undergo a dramatic morphological transition with time, from fibers to bundled structures, fan-shaped sheets, and finally dots, as revealed by AFM imaging of the eluted solution taken at regular intervals of time (Fig. 17D). In addition, evidence from IR analysis (Fig. 17C) suggested that the main intermolecular interaction in the evolving assemblies switches from π–π stacking to hydrogen bonding along with morphological changes. In this example, microfluidic mixing allows the formation of an energetically unfavourable self-assembled structure under kinetic control, which once off-chip relaxes in a downfield manner to its thermodynamically stable state through a series of metastable intermediates. This example also reveals that microfluidic technologies are a powerful tool for unveiling and studying pathway complexity in supramolecular polymerization.

**Fig. 18:**
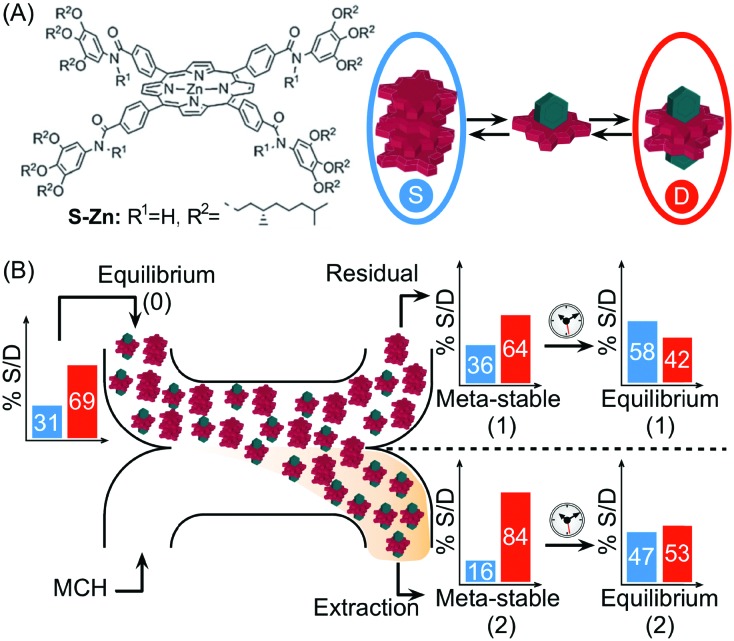
(A) Chemical structure of the (*S*)-Zn–porphyrin, and equilibrium between stacks, S (in the blue circle), and dimers, D (in the red circle), in the presence of pyridine (green polyhedron). (B) Schematic representation of the H-cell used. The differential diffusion of the different species results in different stack-to-dimer ratios as detected at the residual and extraction stream. These correspond to two metastable compositions of the system which equilibrate with time to two different thermodynamic states. Adapted from ref. 41 with permission from The Royal Society of Chemistry.

In another example, Meijer and co-workers exploited continuous flow microfluidics to control the distribution of kinetically metastable porphyrin aggregates in a multicomponent system, based on the different diffusivities of the involved species.^41^ Namely, the (*S*)-Zn–porphyrin in Fig. 18A self-assembles in methylcyclohexane (MCH), forming helical stacks held by strong 4-fold intermolecular H-bonds. However, the addition of pyridine (Pyr) promotes the formation of hydrogen bonded pyridine-capped dimers due to the complexation of Pyr with zinc (Fig. 18A). When an equilibrium mixture of stacks and dimers is eluted against pure MCH in a microfluidic H-cell, a local perturbation of the equilibrium occurs due to the different extent of diffusion of the involved species. Namely, only small dimers and Pyr diffuse significantly into the extraction stream during the residence time on chip (controlled by the flow rate), but not the stacks (Fig. 18B). The latter results in different stack-to-dimer (S/D) ratios at extraction as detected by in-line UV-vis measurements, which correspond to two different metastable compositions of the system. In fact, the S/D ratio in the solutions coming out from the two streams changed with time after collection, evidencing their slow relaxation to (new) equilibrium states (Fig. 18B). This example clearly shows how the microfluidic approach can be used to alter the thermodynamic state of a self-assembled system, by driving the latter transiently out of equilibrium, in a way that is simply not possible using flask-based methodologies.

**Fig. 19:**
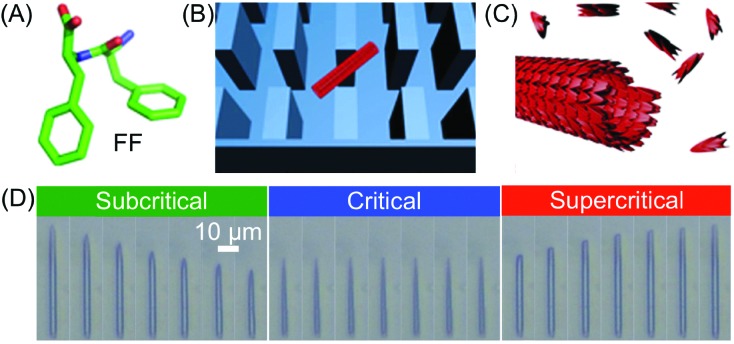
(A) Chemical structure of FF. (B) Schematic illustration of a single FF tube trapped between the micro-scale pillars. (C) Illustration of the FF nanotube growth. (D) Imaging of FF nanotubes at different times under subcritical (1.6 mM, 10 s interval), critical (2.43 mM, 60 s interval), and supercritical (3.20 mM, 100 s interval) concentration conditions. Adapted from ref. 42 with permission from Springer Nature, copyright 2016.

The unsurpassed advantages and suitability of microfluidic devices in controlling supramolecular polymerization, as well as in gaining deep mechanistic insights, have been recently highlighted in the work of Knowles, Gazit and co-workers. By combining a microfluidic method to isolate supramolecular assemblies with real-time optical microscopy, the authors were able to directly visualize *in situ* self-assembly kinetics and dynamics at the level of a single aggregate.^42^ Specifically, a PDMS microfluidic chip incorporating micron-scale pillars was used to trap individual peptide nanotubes formed from the well-known diphenylalanine (FF) building block (Fig. 19A–C). The FF tubes were then subjected to monomer flows of various concentrations (above or below a defined saturation level), which determined their growth or shrinkage (Fig. 19D). Remarkably, the authors found that FF tubes assemble or disassemble through an unexpected unidirectional mechanism, and on the basis of the real-time monitoring of assembly/disassembly, they were able to develop a kinetic model for growth. It should however be stressed that for flask-based syntheses (quiescent conditions) the free building blocks and nanotubes reach a dynamic equilibrium state, in which the on-off rates of binding are equal. Conversely, in a microfluidic device the nanotubes are subjected to a continuous flow of monomers, and thus equilibrium is never reached. Put simply, the dissociation of building blocks from the nanotube (below the critical concentration) does not increase the free monomer concentration of the solution, and hence, the structures continue to shorten until they are completely degraded. Analogously, the incorporation of building blocks above a critical concentration does not deplete monomers from solution and continuous growth occurs. In other words, continuous flow microfluidic devices enable the setting and maintenance of non-equilibrium growth conditions. In another investigation, Knowles and Gazit further demonstrated^43^ that at certain supersaturation levels, radial growth of FF nanotubes can also become significant, along with axial growth. Accordingly, they were able to use a similar microfluidic reactor to achieve non-equilibrium growth of FF crystals with tuneable aspect ratios, in which the crystal aspect ratio is not determined by the equilibrium solubility of each crystal face, but rather by the respective growth rates.

**Fig. 20:**
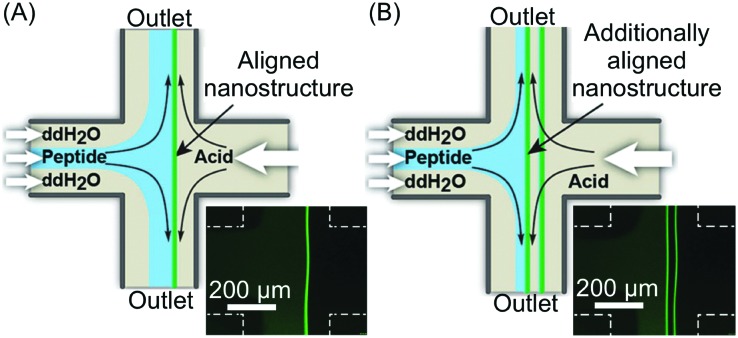
(A) Schematic illustration of the fluidic-directed synthesis of 1D peptide–OPV fibers by using planar extensional flow, and fluorescence microscopy micrograph showing the emitting fiber grown at the reactive interface. (B) Schematic illustration and micrograph showing the formation of two aligned fibers obtained by modulating the flow rates, *i.e.* changing the position of the reactive interface. Adapted from ref. 44 with permission from Wiley-VCH Verlag GmbH & Co., copyright 2013.

In the examples presented so far, the unique hydrodynamic properties of laminar co-flowing streams have been exploited to achieve precise spatio-temporal control over self-assembling events, resulting in the selective formation of non-equilibrium structures under kinetic control, which are normally inaccessible when using flask-based methodologies. However, despite the possible anisotropic growth and aggregate orientation under laminar flow conditions, simple co-flowing streams preclude strong nanostructure alignment, because of the uniform fluid velocity profile. On the other hand, Schroeder, Wilson and co-workers demonstrated the fluid-directed fabrication of highly aligned supramolecular structures from peptide–OPV conjugates, using planar extensional flow (Fig. 20).^44^ Here, the two reactant streams flow in opposite directions towards each other, while the two outlets emerge orthogonally from their cross point (Fig. 20A). Such a microfluidic approach allowed the induction of an unprecedented alignment in the hierarchical OPV fibers (from nano- to mesoscale) due to the extensional/compressional flow characteristics.^44^ The authors used a cross-slot microfluidic device to generate the planar extensional flow, and could directly visualize the formation of 1D peptide–OPV fibers at the interface between a flow-focused, pH buffered monomer stream and an acidic stream (Fig. 20A). Self-assembly is triggered by the protonation of charged amino acid residues and consequent formation of beta sheet-like hydrogen-bonding, and leads to emitting assemblies, easily detectable by fluorescence microscopy (Fig. 20A). Moreover, by modulating the flow rates, the position of the reactive interface at the microchannel junction could be systematically varied, which allows the formation of multiple parallelly aligned nanostructures or their subsequent disassembly (Fig. 20B). The spatial control over self-assembly is relevant to the integration of π-conjugated supramolecular materials into electronic devices. Finally, polarized and time resolved fluorescence spectroscopy confirmed a strong supramolecular alignment of the OPV stacks within the fibers and along the direction of the flow, which form an extended exciton (ordered structure). In this work, the authors showed that a planar extensional flow is an ideal tool for aligning supramolecular aggregates, since, unlike laminar co-flows (with almost uniform velocity profiles), it intrinsically provides a velocity gradient along the flow direction.

### Supramolecular chemistry in segmented flow microfluidics

**Fig. 21:**
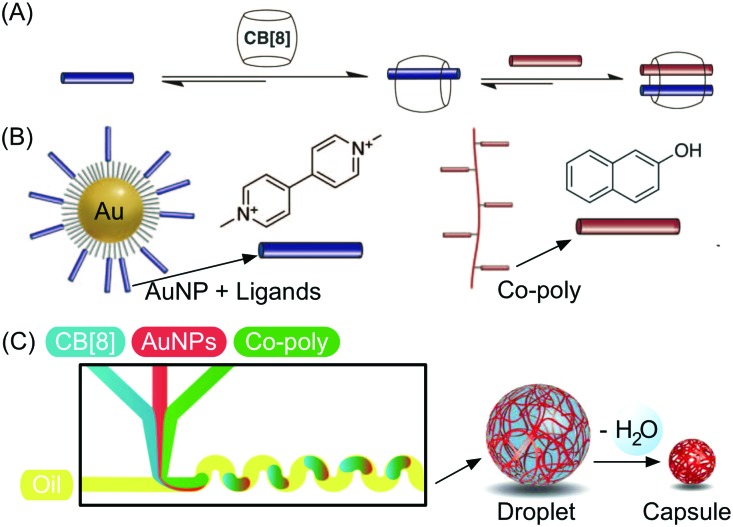
(A) Illustration of the formation of a ternary inclusion complex between the host CB[8] and two different planar guests. (B) Schematic representation of the Au nanoparticles functionalized with methylviologen (guest) and of the copolymer functionalized with naphthol moieties (guest). (C) Schematic representation of the microdroplet generation process using a microfluidic T-junction device, showing the wiggled channel used for rapid mixing of reactant solution, and schematic representation of the microcapsule formation and of the following dehydration step. Adapted from ref. 46 with permission from The American Association for the Advancement of Science, copyright 2012.

**Fig. 22:**
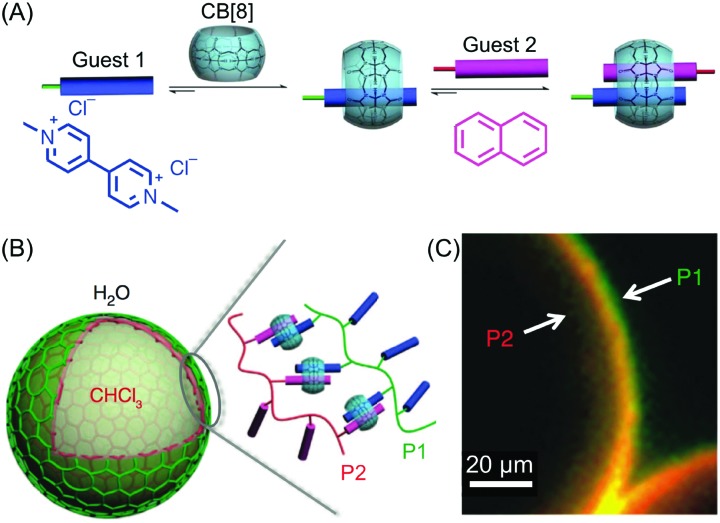
(A) Illustration of the formation of the ternary inclusion complex between the host CB[8] and two different guests. Adapted from ref. 45 with permission from American Chemical Society, copyright 2017. (B) Schematic representation of the supramolecular assembly of the two polymers P1 and P2 at the interface of chloroform-in-water droplets. (C) Fluorescence micrographs demonstrating the interfacial assembly of P2 (Rhodamine-tagged, red) within the droplet and P1 (Fluorescein-tagged, green) present in the external media. Adapted from ref. 47 with permission from Springer Nature, copyright 2014.

Besides studies based on continuous flow microfluidics, supramolecular chemistry has also been performed within segmented flows for the preparation of non-covalent architectures, such as microcapsules. For example, Scherman and co-workers investigated supramolecular polymer microcapsules assembled at the liquid–liquid interface of microfluidic droplets by host–guest chemistry, specifically cucurbit[n]uril (CB[*n*])-mediated host–guest interactions (Fig. 21A).^45^ In a recent work,^46^ they exploited the tendency of colloidal particles to accumulate at the interface between immiscible liquids, so as to reduce the interfacial energy (so called Pickering emulsions) and drive the localized formation of a supramolecular polymer network based on a ternary inclusion complex (Fig. 21A). Specifically, a droplet-based microfluidic approach (a T-junction microfluidic device) was used to generate water microdroplets in an oil carrier phase (Fig. 21C). The aqueous phase contained CB[8] (the host), gold nanoparticles decorated with methylviologen (AuNPs), and a copolymer functionalized with naphthol moieties (Co-poly), where methylviologen and naphthol are the two guest moieties (Fig. 21B). Migration at the droplet interface allows gold nanoparticles to pull the copolymer, due to the favourable host–guest complexation, and as a result a supramolecular shell is formed at the water/oil interface leading to the formation of microcapsules (Fig. 21C). Remarkably, the microcapsules retain their spherical empty structure after evaporation of the water droplet and can be easily loaded with a cargo alongside their generation. In another example,^47^ chloroform-in-water microdroplets were used to template the formation of supramolecular microcapsules by interfacial self-assembly (Fig. 22). Here, two polymers bearing different guest moieties were dissolved: one in the aqueous continuous phase, together with the CB[8] host (P1), and the other delivered in chloroform microdroplets (P2) (Fig. 22B). In this case, the formation of the ternary host–guest complex across the liquid–liquid droplet interface drove the formation of a supramolecular polymeric shell, resulting in robust microcapsules, as evidenced by fluorescence microscopy (Fig. 22C). As a result of their non-covalent dynamic nature, supramolecular microcapsules based on host–guest complexation have also been elegantly engineered to be responsive to external stimuli (such as reducing agents and light), which allows for controlled release of the encapsulated cargos.

## 4. Conclusions and future prospects

In this review, we have hopefully shown that the unique conditions offered by microfluidic reactors can be used to control the self-assembly of a range of materials, in solution and at a surface (Section 2), and also, supramolecular processes in solution (Section 3). Whereas common processing approaches (and chemical strategies) fail to provide precise control over pathway selection to non-equilibrium assemblies (either kinetically trapped or metastable states) or thermodynamic structures, recent studies clearly demonstrate that microfluidic devices are extraordinarily effective toolbox technologies to unveil, study and design rational pathway selection mechanisms. When using microfluidic devices, pathway selection (towards a desired property and function) can be potentially achieved in molecular-based assemblies. Therefore, we believe that microfluidic mixing is inducing a revolutionary paradigm shift in self-assembly research and supramolecular chemistry, and thus, in the preparation of materials and devices. For example, it has been recently demonstrated that microfluidic devices can enable the formation of artificial microfibers (of a self-assembled material) that not only mimic the morphology of living tissues but also their function.^48^ Indeed, it is envisioned that microfluidic technologies will become a fundamental and indispensable tool in the isolation and study of non-equilibrium assemblies, and will undoubtedly play a major role in the fabrication of new functional materials that are currently inaccessible.

However, there are still challenges in this nascent research field that must be considered and resolved. First, it is important to be able to monitor supramolecular interactions in real-time and on-chip, a facility that will enable an excellent understanding of structure–property relationships and a selective collection (isolation) of intermediate assemblies and structures. Indeed, in all the studies presented above, the characterization of the structures generated inside microfluidic devices is always done off-line (off-chip) using traditional imaging or analytical tools, such as transmission electron microscopy (TEM), scanning electron microscopy (SEM), atomic force microscopy (AFM), Fourier-transform infrared spectroscopy (FT-IR), thermogravimetric analysis (TGA), and elemental analysis. Actually, techniques such as on-chip (confocal) Raman spectroscopy^35^ and on-chip X-ray diffraction studies^49^ are able to circumvent the problems associated with on-chip real-time monitoring. These techniques can be used to provide information about the chemical composition or structure (*e.g.* crystallinity and/or polymorphism) of the materials generated inside the chip and in real-time, but do not give detailed information of the mechanisms and processes occurring during their self-assembly. Recently, for example, the thermodynamics and kinetics of supramolecular aggregation processes induced by hydrophobic interactions have been studied in real-time (and on sub-microsecond time scales) employing a microfluidic-based platform.^50^ Fluorescence measurements are still the most frequently used detection methods in microfluidic devices, usually being more selective and sensitive than other optical techniques. However, in this example, aggregation-induced emission of the supramolecular assemblies under investigation is required to obtain significant information, which in turn hinders the extension of this microfluidic approach to other systems and materials.

Further challenges include precise temperature control in microfluidic devices and the ability to adjust the chemical and physical properties of the materials used for the fabrication of the microfluidic devices themselves. Advancements in these areas will enable access to a wider range of conditions, and ultimately will expand the frontiers of materials engineering and supramolecular chemistry. Indeed, we believe that these challenges will be overcome in the near future as more researchers, from diverse fields and with different expertise, adopt microfluidic technologies to study chemical and physical phenomena that cannot be investigated and resolved by other methods.

## Conflicts of interest

There are no conflicts to declare.

## References

[cit1] Amabilino D. B., Veciana J. (2006). Top. Curr. Chem..

[cit2] Lehn J.-M., Supramolecular Chemistry: Concepts and Perspectives, Wiley, 1995.

[cit3] Mann S. (2009). Nat. Mater..

[cit4] Fialkowski M., Bishop K. J. M., Klajn R., Smoukov S. K., Campbell C. J., Grzybowski B. A. (2006). J. Phys. Chem. B.

[cit5] Clapham D. E. (2007). Cell.

[cit6] Grzybowski B. A., Fitzner K., Paczesny J., Granick S. (2017). Chem. Soc. Rev..

[cit7] Lovrak M., Hendriksen W. E., Maity C., Mytnyk S., van Steijn V., Eelkema R., van Esch J. H. (2017). Nat. Commun..

[cit8] Noorduin W. L., Grinthal A., Mahadevan L., Aizenberg J. (2013). Science.

[cit9] Elvira K. S., Solvas X. C. I., Wootton R. C., deMello A. J. (2013). Nat. Chem..

[cit10] Zang H.-Y., De La Oliva A. R., Miras H. N., Long D.-L., McBurney R. T., Cronin L. (2014). Nat. Commun..

[cit11] Hassan N., Cabuil V., Abou-Hassan A. (2013). Angew. Chem., Int. Ed..

[cit12] Beatus T., Shani I., Bar-Ziv R. H., Tlusty T. (2017). Chem. Soc. Rev..

[cit13] Hou X., Zhang Y. S., Trujillo-de Santiago G., Alvarez M. M., Ribas J., Jonas S. J., Weiss P. S., Andrews A. M., Aizenberg J., Khademhosseini A. (2017). Nat. Rev. Mater..

[cit14] Sorrenti A., Leira-Iglesias J., Markvoort A. J., de Greef T. F. A., Hermans T. M. (2017). Chem. Soc. Rev..

[cit15] Coskun A., Spruell J. M., Barin G., Dichtel W. R., Flood A. H., Botros Y. Y., Stoddart J. F. (2012). Chem. Soc. Rev..

[cit16] Segura J. L., Martín N. (2001). Angew. Chem., Int. Ed..

[cit17] Puigmartí-Luis J., Laukhina E. E., Laukhin V. N., Pérez del Pino Á., Mestres N., Vidal-Gancedo J., Rovira C., Amabilino D. B. (2009). Adv. Funct. Mater..

[cit18] Puigmartí-Luis J., Minoia A., Lei S., Geskin V., Li B., Lazzaroni R., De Feyter S., Amabilino D. B. (2011). Chem. Sci..

[cit19] Puigmartí-Luis J., Schaffhauser D., Burg B. R., Dittrich P. S. (2010). Adv. Mater..

[cit20] Knight J. B., Vishwanath A., Brody J. P., Austin R. H. (1998). Phys. Rev. Lett..

[cit21] Squires T. M., Quake S. R. (2005). Rev. Mod. Phys..

[cit22] Lu M., Yang S., Ho Y.-P., Grigsby C. L., Leong K. W., Huang T. J. (2014). ACS Nano.

[cit23] Amstad E., Gopinadhan M., Holtze C., Osuji C. O., Brenner M. P., Spaepen F., Weitz D. A. (2015). Science.

[cit24] Rubio-Martinez M., Imaz I., Domingo N., Abrishamkar A., Mayor T. S., Rossi R. M., Carbonell C., deMello A. J., Amabilino D. B., Maspoch D., Puigmartí-Luis J. (2016). Adv. Mater..

[cit25] Rodríguez-San-Miguel D., Abrishamkar A., Navarro J. A., Rodriguez-Trujillo R., Amabilino D. B., Mas-Ballesté R., Zamora F., Puigmartí-Luis J. (2016). Chem. Commun..

[cit26] Rodríguez-San-Miguel D., Corral-Pérez J. J., Gil-González E., Cuellas D., Arauzo J., Monsalvo V. M., Carcelén V., Zamora F. (2017). CrystEngComm.

[cit27] Ameloot R., Vermoortele F., Vanhove W., Roeffaers M. B., Sels B. F., De Vos D. E. (2011). Nat. Chem..

[cit28] Furukawa S., Reboul J., Diring S., Sumida K., Kitagawa S. (2014). Chem. Soc. Rev..

[cit29] Faustini M., Kim J., Jeong G.-Y., Kim J. Y., Moon H. R., Ahn W.-S., Kim D.-P. (2013). J. Am. Chem. Soc..

[cit30] Witters D., Vergauwe N., Ameloot R., Vermeir S., De Vos D., Puers R., Sels B., Lammertyn J. (2012). Adv. Mater..

[cit31] Liu X., Yi Q., Han Y., Liang Z., Shen C., Zhou Z., Sun J., Li Y., Du W., Cao R. (2015). Angew. Chem., Int. Ed..

[cit32] Puigmartí-Luis J., Rubio-Martínez M., Imaz I., Cvetkovic B. Z., Abad L., Pérez del Pino A., Maspoch D., Amabilino D. B. (2013). ACS Nano.

[cit33] Puigmartí-Luis J., Paradinas M., Bailo E., Rodriguez-Trujillo R., Pfattner R., Ocal C., Amabilino D. B. (2015). Chem. Sci..

[cit34] Hou S., Wang S., Yu Z. T., Zhu N. Q., Liu K., Sun J., Lin W.-Y., Shen C. K.-F., Fang X., Tseng H.-R. (2008). Angew. Chem..

[cit35] Lee S. G., Lee H., Gupta A., Chang S., Doyle P. S. (2016). Adv. Funct. Mater..

[cit36] Numata M., Nishino Y., Sanada Y., Sakurai K. (2015). Chem. Lett..

[cit37] Numata M., Kozawa T., Nogami R., Tanaka K., Sanada Y., Sakurai K. (2015). Bull. Chem. Soc. Jpn..

[cit38] Numata M., Kozawa T. (2013). Chem. – Eur. J..

[cit39] Sorrenti A., Rodriguez-Trujillo R., Amabilino D. B., Puigmartí-Luis J. (2016). J. Am. Chem. Soc..

[cit40] Numata M., Sato A., Nogami R. (2015). Chem. Lett..

[cit41] Helmich F., Meijer E. W. (2013). Chem. Commun..

[cit42] Arnon Z. A., Vitalis A., Levin A., Michaels T. C., Caflisch A., Knowles T. P., Adler-Abramovich L., Gazit E. (2016). Nat. Commun..

[cit43] Mason T. O., Michaels T. C. T., Levin A., Gazit E., Dobson C. M., Buell A. K., Knowles T. P. J. (2016). J. Am. Chem. Soc..

[cit44] Marciel A. B., Tanyeri M., Wall B. D., Tovar J. D., Schroeder C. M., Wilson W. L. (2013). Adv. Mater..

[cit45] Liu J., Lan Y., Yu Z., Tan C. S. Y., Parker R. M., Abell C., Scherman O. A. (2017). Acc. Chem. Res..

[cit46] Zhang J., Coulston R. J., Jones S. T., Geng J., Scherman O. A., Abell C. (2012). Science.

[cit47] Zheng Y., Yu Z., Parker R. M., Wu Y., Abell C., Scherman O. A. (2014). Nat. Commun..

[cit48] Onoe H., Okitsu T., Itou A., Kato-Negishi M., Gojo R., Kiriya D., Sato K., Miura S., Iwanaga S., Kuribayashi-Shigetomi K., Matsunaga Y. T., Shimoyama Y., Takeuchi S. (2013). Nat. Mater..

[cit49] Khvostichenko D. S., Schieferstein J. M., Pawate A. S., Laible P. D., Kenis P. J. (2014). Cryst. Growth Des..

[cit50] Jiang L., Cao S., Cheung P. P.-H., Zheng X., Leung C. W. T., Peng Q., Shuai Z., Tang B. Z., Yao S., Huang X. (2017). Nat. Commun..

